# Can we escape from top-priority ESKAPE pathogens?

**DOI:** 10.1080/22221751.2026.2614739

**Published:** 2026-01-08

**Authors:** Lingbing Zeng, Youjun Feng, Minggui Wang

**Affiliations:** a Department of Laboratory Medicine and Branch of Gaoxin, The First Affiliated Hospital, Jiangxi Medical College, Nanchang University, Nanchang, People’s Republic of China; b Key Laboratory of Multiple Organ Failure (Ministry of Education), Departments of Microbiology and General Intensive Care Unit of the Second Affiliated Hospital, Zhejiang University School of Medicine, Hangzhou, People’s Republic of China; c Key Laboratory of Clinical Pharmacology of Antibiotics, Institute of Antibiotics, Huashan Hospital, Fudan University, National Heath Commission of the People's Republic of China, Shanghai, People’s Republic of China

**Keywords:** ESKAPE pathogens, antimicrobial resistance, surveillance, treatment, new drug

## Abstract

The ESKAPE pathogens – *Enterococcus faecium*, *Staphylococcus aureus*, *Klebsiella pneumoniae*, *Acinetobacter baumannii*, *Pseudomonas aeruginosa*, and *Enterobacter spp.* – are designated by the World Health Organization as critical-priority multidrug-resistant organisms. These bacteria are leading contributors to the global antimicrobial resistance crisis, significantly increasing morbidity, mortality, and healthcare costs worldwide. Their capacity to evade conventional antibiotics continues to complicate clinical management and undermine infection control efforts. Tackling the global threat of ESKAPE pathogens demands coordinated and sustained interventions. This mini review summarizes recent evidence on the burden and prevalence of ESKAPE infections and assesses emerging strategies to combat resistance. Progress in surveillance and promising preclinical and clinical studies of novel therapies underscore that integrated approaches are crucial. Moving forward, a balanced emphasis on prevention, surveillance, and therapeutic innovation will be essential to mitigating the threat posed by ESKAPE pathogens over the coming decade.

## Main text

Antimicrobial resistance (AMR) poses a significant public health challenge globally, with considerable negative effects on both health and the economy. It is estimated that 1.14 million deaths were attributable to bacterial AMR, and 4.71 million deaths were associated with bacterial AMR in 2021. Moreover, the mortality rate due to AMR escalated by over 80% among individuals aged 70 and above from 1990 to 2021 [1]. The data from China exhibited a similar trend, with 160,268 deaths (representing 14% of the total number of deaths recorded globally) attributable to bacterial AMR, along with a further 711,855 deaths (15% of the global total) associated with bacterial AMR [1,2]. A similar increase was observed in the death rate among individuals over the age of 65, which rose by 68% from 1991 to 2021 in China [2]. To address the AMR crisis, the World Health Organization (WHO) launched the Global Action Plan on Antimicrobial Resistance in 2015, urging countries to adopt strong measures to manage AMR, and established the Bacterial Priority Pathogens List (BPPL), which was renewed in 2024 [3,4]. ESKAPE pathogens – *Enterococcus faecium*, *Staphylococcus aureus*, *Klebsiella pneumoniae*, *Acinetobacter baumannii*, *Pseudomonas aeruginosa*, and *Enterobacter spp.* – have been identified as critical multidrug-resistant bacteria and are listed with “priority status” in the BPPL [5]. In China, where ESKAPE pathogens were responsible for 117,403 (73%) of the 160,268 deaths attributable to bacterial AMR [2]. In addition, the acquisition of antimicrobial resistance genes by ESKAPE pathogens has reduced the treatment options for serious infections and increased the disease burden and mortality rates due to treatment failure. In this mini review, we discuss the epidemiology of ESKAPE pathogens with a focus on China and worldwide, as well as potential new strategies for the treatment of ESKAPE pathogens in future.

## Major concerns surrounding ESKAPE pathogens

### Vancomycin-resistant Enterococcus faecium (VREF)

**Figure 1.**
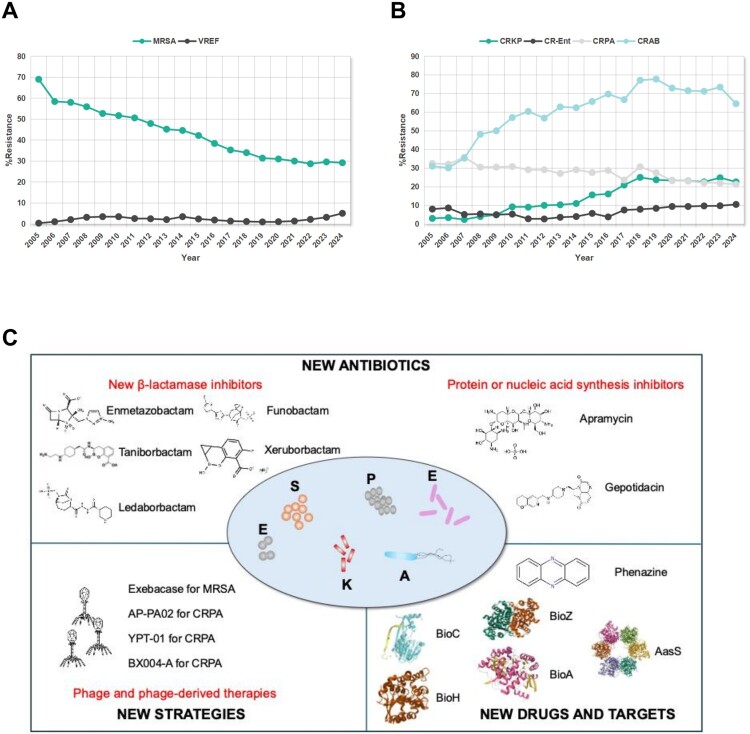
Antimicrobial resistance profiles of ESKAPE pathogens in China and strategies to overcome ESKAPE-associated infections.

*E. faecium* is common in the human gastrointestinal microbiota and exhibits intrinsic resistance to cephalosporins*.* Recently, the prevalence of VREF has been increasing on a global scale, with its distribution varying significantly among different regions. Western Europe has a lower proportion, ranging from 0.3% to 3%, while in parts of Southern and Eastern Europe, this proportion may exceed 50%, as is also the case in the United States and Latin America (https://atlas-surveillance.com). In China, although the proportion of VREF is below 5% according to the national surveillance network CHINET (www.chinets.com) (Figure 1(A)), notably lower compared to most other countries [6]. Please note most resistance data in this mini review were cited from CHINET and referred to our previous review in JAC-Antimicrobial Resistance [6], but the data are updated from 2022 to 2024. The prevalence of VREF has increased significantly recently, which was over 5% in 2024 from CHINET data. The CARSS surveillance data showed that the proportions of VREF in Beijing and Guangdong were 13.1% and 22.6%, respectively, in 2024 (https://www.carss.cn/). The prevalence of VREF increased in hospitals in Guangdong province from <5% before 2021 to 20-50% in 2023, which was related to outbreak of an emerging clone ST80 [7].

The absence of oral vancomycin in China could contribute to the low prevalence of VREF [6], but vancomycin oral capsules had been launched in China for clinical use in July 2024, therefore the prevalence of VREF needs to be closely monitored afterwards. Molecular epidemiological analysis has revealed that clonal complex 17, sequence type (ST) 80 has quickly become the dominant VREF lineage in most countries (Table 1) [8]. *VanA* and *vanM* are the two dominant genotypes associated with VREF globally.

**Table 1.** Characteristics of ESKAPE pathogens and treatment options.

ESKAPE pathogen	Prevalent clone	Current antimicrobial	New antimicrobial or strategy	
			New antimicrobial	Category or Antibacterial class
Vancomycin-resistant *Enterococcus faecium*	ST17, ST80, ST117, ST412, ST584, ST664, ST736	Daptomycin, Linezolid, Tigecycline, Eravacycline, Omadacycline, Oritavancin	Contezolid	Oxazolidinones
			Reltecimod	CD28 T cell receptor mimetic
Methicillin-resistant *Staphylococcus aureus*	ST59, ST8	Vancomycin, Daptomycin, Linezolid, Ceftaroline, Ceftobiprole, Trimethoprim–sulfamethoxazole, Delafloxacin, Oritavancin, Dalbavancin, Telavancin	Contezolid	Oxazolidinones
			Gepotidacin	Triazaacenaphthylene
			Exebacase	Phage
			Suvratoxumab	Monoclonal antibody against *S. aureus*
			Tosatoxumab	Monoclonal antibody against *S. aureus*
			Reltecimod	CD28 T cell receptor mimetic
Carbapenem-resistant *Klebsiella pneumoniae*	ST11, ST258, ST512	Ceftazidime–avibactam, Meropenem–vaborbactam, Imipenem–cilastatin–relebactam, Cefiderocol, Tigecycline, Eravacycline, Polymyxin B	Cefepime- taniborbactam	β-lactamase inhibitor combination
			Cefepime- zidebactam	β-lactamase inhibitor combination
			Reltecimod	CD28 T cell receptor mimetic
Carbapenem-resistant *Acinetobacter baumannii*	ST2, ST1, ST3	Sulbactam–durlobactam, Cefiderocol, Minocycline, Tigecycline, Eravacycline, Polymyxin B	Cefepime- zidebactam	β-lactamase inhibitor combination
** **			Reltecimod	CD28 T cell receptor mimetic
Carbapenem-resistant *Pseudomonas aeruginosa*	ST235, ST111, ST175, ST463	Ceftolozane–tazobactam, Ceftazidime–avibactam, Imipenem–cilastatin–relebactam, Cefiderocol, Polymyxin B	Cefepime- taniborbactam	β-lactamase inhibitor combination
** **			Cefepime- zidebactam	β-lactamase inhibitor combination
** **			Reltecimod	CD28 T cell receptor mimetic
Carbapenem-resistant *Enterobacter spp.*	ST114, ST171	Ceftazidime–avibactam, Meropenem–vaborbactam, Imipenem–cilastatin–relebactam, Cefiderocol, Tigecycline, Eravacycline, Polymyxin B	Cefepime- taniborbactam	β-lactamase inhibitor combination
** **			Cefepime- zidebactam	β-lactamase inhibitor combination
** **			Reltecimod	CD28 T cell receptor mimetic

### Methicillin-resistant Staphylococcus aureus (MRSA)

*S. aureus* is a significant human pathogen that can cause various infections. MRSA’s ability to adapt to different conditions has the potential to complicate infection management. The prevalence of MRSA is stable in North and Latin America, at 37.3% and 41.9% in 2023, respectively (https://atlas-surveillance.com). In contrast, there is a remarkable decreasing prevalence of MRSA in China, from 69% in 2005 to 29.2% in 2024, according to CHINET surveillance (Figure 1(A)).

Molecular epidemiology studies demonstrated that community-associated MRSA (CA-MRSA) differs from healthcare-associated MRSA (HA-MRSA). CA-MRSA typically harbours type IV or V staphylococcal chromosomal cassette mec (SCCmec) elements, and in some strains, Panton–Valentine leucocidin (PVL). Conversely, HA-MRSA carries SCCmec I–III [9]. However, in recent years, CA-MRSA strains have emerged in healthcare settings, thus altering the epidemiology of MRSA.

Multi-locus sequence typing results indicate that the predominant strain of MRSA has shifted from ST239 to ST59 in recent years, which might be one of the key factors contributing to the decline in MRSA prevalence in China. Meanwhile, ST8 has been consistently predominant in the USA [8]. During this period, Chinese hospitals implemented various preventive measures, such as hand hygiene measures, which may have led to the decrease in ST239. The more resilient ST59 strain has gradually gained dominance (Table 1). In contrast, after the highly virulent strain ST398 acquired antibiotic resistance genes and became MRSA, its virulence did not undergo significant changes. This is an area that requires close monitoring in the future.

### Carbapenem-resistant *Klebsiella pneumoniae* (CRKP)

The prevalence of CRKP has dramatically increased over the last decade. Data indicates that the CRKP prevalence (imipenem resistance) rose from 2.9% to around 22.6% in China during 2005–2024 (Figure 1(B)). From 2008 to 2018, it increased from 2.5% to 25%, indicating an over 10-fold surge over the decade (Figure 1(B)). Fortunately, it decreased slightly to 22.6% in 2024. ST11 strains carrying carbapenemase genes (especially *bla*_KPC-2_) are endemic to China, and they remain significantly more prevalent worldwide compared to ST258, notably in Europe and the US (Table 1). Polymyxin is a last resort against severe infections caused by multidrug-resistant Gram-negative pathogens, especially CRKP. However, polymyxin resistance of CRKP reached a rate of 11.2% in China in 2024 (www.chinets.com). Polymyxin resistance mainly occurs due to 4’-phosphoethanolamine (PEA) modification of lipid A on LPS, which can be caused by chromosome-encoded machinery (such as *mgrB*) or plasmid-transferred mobilized colistin resistance (*mcr* genes) [10].

Recently, the emergence of both carbapenem-resistant and hypervirulent *K. pneumoniae*, which exhibits resistance to most clinically available β-lactams, has been of particular concern, significantly limiting treatment options. This kind of bacteria can emerge through two different ways: hypervirulent strains acquire carbapenem-resistant plasmid (CR-hvKP), or carbapenem-resistant strains acquire hypervirulent plasmid (hv-CRKP). The IncFIIK34 KPC plasmid can overcome the protective barriers of the thick capsule and hypermucoviscosity characteristic of hypervirulent *K. pneumoniae* (hvKP; primarily ST23, ST86, and ST65), enabling plasmid acquisition and the expression of carbapenem resistance within these strains [11]. In addition, IncFIB(Mar)-type conjugative plasmids can assist to transfer highly virulent plasmids into carbapenem-resistant strains (mainly the ST11 type) [12]. The emergence and spread of CR-hvKP and hv-CRKP pose a significant threat to clinical practice and public health worldwide. Early diagnosis and routine surveillance could be effective measures to control these infections.

### Carbapenem-resistant *Acinetobacter baumannii* (CRAB)

CRAB strains are frequently linked to outbreaks in intensive care units (ICUs), posing a severe challenge to infection control in healthcare facilities. Data from CHINET shows that the proportion of *A. baumannii* strains resistant to imipenem increased from 30.1% in 2005 to 64.5% in 2024 (Figure 1(B)). Over a 31-year period, this pathogen has demonstrated the third most rise in attributable deaths, exceeding 100,000 in China [2]. This trend highlights CRAB as a critical driver of the AMR burden in China. Multilocus sequence typing (MLST) analyses show that most clinical isolates belong to ST2 type, comprising roughly 59% of CRAB worldwide (Table 1). Carbapenemase OXA-23 accounts for most CRAB cases worldwide; 99% of CRAB strains in China produce OXA-23. The treatment options for CRAB are very limited, with only polymyxin and tigecycline available [13]. Fortunately, new antibiotics have recently been launched, including sulbactam-durlobactam and eravacycline. However, the monitoring and treatment of CRAB remain key issues that require continued attention.

### Carbapenem-resistant Pseudomonas aeruginosa (CRPA)

*P. aeruginosa* is an opportunistic pathogen responsible for persistent infections. It has several inherent resistance mechanisms, such as barriers to drug permeability, a variety of multidrug efflux pumps, and a chromosomally encoded AmpC enzyme. Additionally, *P. aeruginosa* produces multiple virulence factors, with type III secretion system (T3SS) effectors being particularly significant. CHINET surveillance data indicates that the proportion of *P. aeruginosa* strains resistant to imipenem decreased from 32.5% in 2005 to 21.3% in 2024 (Figure 1(B)). Although most CRPA strains occur due to the loss of the outer membrane porin OprD and overexpression of the MexAB–OprM efflux pump, carbapenemase-producing strains have been increasing recently [14]. A cohort study revealed that the prevalence of carbapenemases in CRPA varies across different regions: in South and Central America, it is 69% (of 127 tested); in Australia and Singapore, 57% (of 56); in China, 32% (of 171); in the Middle East, 30% (of 91); and in the USA, 2% (of 527) (Table 1) [14]. KPC-2 and VIM-2 are the most common carbapenemases. Plasmids, as mobile genetic elements, play a crucial role in transferring KPC-2 in carbapenemase- producing CRPA (CP-CRPA), particularly the *IS*Kpn6-*bla*_KPC-2_-*IS*Kpn27-associated plasmid [15].

Many challenges are encountered in the treatment of CP-CRPA. First, the production of carbapenemases leads to resistance to many antibiotics, especially when metalloenzymes are present, which results in resistance to various novel β-lactam–β-lactamase inhibitor combinations. Second, CP-CRPA is not only resistant to carbapenems, but also shows increasing resistance to other antibiotics, leading to a gradual increase in the proportion of “difficult-to-treat resistant” *P. aeruginosa* (DTR-PA) among CP-CRPA. This indicates the need for real-time monitoring of CP-CRPA and the development of novel therapeutic drugs and strategies.

### Carbapenem-resistant *enterobacter spp.* (CR-Ent)

The epidemiological trends of carbapenem-resistant CR-Ent exhibit distinct geographical patterns, with significant challenges observed in China compared to other regions. In China, the proportion of *Enterobacter spp.* resistant to imipenem increased from 8% in 2005 to 10.5% in 2024 (Figure 1(B)). Carbapenem resistance greatly impacts the treatment of *Enterobacter spp.* infections and its outcomes. In recent years, most *Enterobacter spp.* have been classified as *Enterobacter cloacae* complexes when using automated microbial identification systems and mass spectrometers [16]. However, whole-genome sequencing has been used to classify *Enterobacter cloacae* complexes as *Enterobacter xiangfengensis*, *Enterobacter hormaechei*, *Enterobacter cloacae,* etc. *Enterobacter xiangfangensis* is the most common human-source CR-Ent in China, with a high proportion of carbapenemase-producing strains compared to other regions. These strains always carry IncX3-type *bla*_NDM_ plasmids. Additionally, ST171 and ST116 *Enterobacter xiangfangensis* are the primary lineages of CR-Ent strains, with ST171 more prevalent in elderly patients (Table 1) [16]. Worryingly, Chinese isolates have exhibited the co-occurrence of multiple resistance genes, always including CR-Ent with *mcr-9/10*. Future research should focus on longitudinal studies to track these dynamics, especially in high-burden regions like China.

## New antibiotics targeting ESKAPE pathogens

As of 2020, a range of novel antibiotics have been introduced for clinical use in China, including ceftobiprole, contezolid, omadacycline, eravacycline, imipenem-relebactam, ceftolozane-tazobactam, sulbactam–durlobactam and aztreonam–avibactam (Table 1). These antibiotics target different ESKAPE pathogens. Ceftobiprole, contezolid, and omadacycline are all potential treatment options for MRSA, while contezolid, omadacycline and eravacycline have been demonstrated to be effective against VREF. Sulbactam-durlobactam is indicated for the treatment of CRAB, while imipenem-relebactam is indicated for the treatment of carbapenem-resistant Enterobacterales (CRE). Aztreonam-avibactam was formally approved by the National Medical Products Administration (NMPA) on 30 June 2025. This antibiotic targets CRE and CRPA and is effective against bacteria that produce serine- and metallo-β-lactamases.

Despite the advent of numerous novel antibiotics employed in clinical settings, these treatments proved ineffective in fully addressing the therapeutic challenges posed by these infections. Most of the above new antimicrobials target a few or only one specific ESKAPE pathogens. Sulbactam-durlobactam is just active against CRAB and is not active for CRAB strains producing metallo-β-lactamases [17]. Ceftolozane-tazobactam only has activity against CRPA. Ceftazidime-avibactam and imipenem-relebactam also exhibit a narrow antimicrobial spectrum with activities against CRE and CRPA producing serine carbapenemases but not the strains producing metallo-beta-lactamase. In the contemporary context, two major challenges have been identified: the limited availability of these medications because of their high costs and/or not covering by medical insurance, and the emergence of resistance mechanisms. Up to 2023, more than 150 *bla*_KPC_ variants have been reported worldwide and most of the new variants were discovered during 2021 to 2023 [18]. The rapid increase of KPC variants may be related to the clinical use of ceftazidime-avibactam and usually mediate resistance to this drug. It is therefore recommended that consistent surveillance and rational use of these antibiotics be implemented in the future. This will ensure that resistance to these antibiotics among ESKAPE pathogens is controlled at a low level.

## Pathways towards escape: innovative strategies are needed

China is among the most rapidly ageing countries worldwide, with the proportion of individuals aged 60 years and over projected to reach 28% by 2040. It is evident that elderly individuals are susceptible to infectious diseases, particularly those caused by ESKAPE pathogens [2]. Mitigating the AMR threat requires a multifaceted strategy. Chinese government has enacted significant policies to combat antimicrobial resistance (AMR), promoting the construction of national and regional AMR surveillance systems (such as CARSS and CHINET) and the rational use of antibiotics in both the medical and agricultural fields. Thus, the VREF in China is lower than 5%, the rate of MRSA has been decreasing over the last twenty years, and the prevalence of CRKP, CRPA and CRAB exhibits a slight decrease in recent five years, indicating that some ESKAPE pathogens can be combatted through interventions.

However, the emergence of CRKP-associated hypervirulence, CP-CRPA and the complicated resistance of CR-Ent have emerged as new problems. Therefore, to escape from ESKAPE pathogens, novel antimicrobial agents and treatment strategies are increasingly required, as microbial resistance mechanisms continue to evolve.

First, new compounds have become an important direction for discovering new antimicrobial agents. Phenazines are a large group of nitrogen-containing heterocyclic compounds that show broad-spectrum antibiotic properties, including against MRSA. Meanwhile, phenazine-inspired antibiotics are specifically effective for biofilm inhibition, providing a potential approach to tackle AMR [19]. Further in vivo evaluations are required to facilitate a more comprehensive understanding of its role as an antimicrobial agent [20]. Zosurabalpin, as a new macrocyclic peptide antibiotic, can block the transport of bacterial lipopolysaccharide and will soon enter into phase III trials. It has antibacterial activity against *A. baumannii*, including CRAB, but not active against other bacteria [21].

Secondly, new targets, especially unique and pathogen-specific candidates, are recognized as vital sources for developing new antimicrobial agents. Biotin and the type II fatty acid synthesis systems are vital for bacterial survival. Many studies have confirmed that biotin synthesis coenzymes (like BioA, BioC, BioH and BioZ) and type II fatty acid synthetases (such as AasS) are vital for bacterial virulence, especially for ESKAPE pathogens, and could be novel targets for antimicrobial drugs [22].

Third, lytic bacteriophages are an important weapon in the post antibiotic era due to their effective treatment of severe bacterial infections. In recent years, personalized phage therapy has been used to treat multidrug-resistant *A. baumannii*, *P. aeruginosa*, and some other ESKAPE pathogens achieving excellent results (Figure 1(C)) [23,24]. However, current challenges to the clinical implementation of phage therapy include its narrow host range, time-intensive natural isolation process, phage resistance, and ecological impacts on commensal microbiota. Solutions have been developed to address the aforementioned issues: the utilization of phage cocktails or the combination of phages with antibiotics to address their narrow spectrum, the genetic editing of phages to circumvent natural isolation, and the implementation of more rigorous evaluation methods to ensure the safety of phage therapy [24]. Therefore, developing new phages is crucial for the future treatment of antibiotic-resistant ESKAPE pathogens.

In summary, combined actions, including national policies, routine surveillance, discovering new drugs and targets, and developing novel treatment strategies, have demonstrated effectiveness in reducing resistance rates and advancing global health efforts against AMR.

## Conclusion

Although the challenges posed by ESKAPE pathogens are complicated, a way to escape from them still exists. We may achieve this through strengthening public health defenses and accelerating research and development into diverse, innovative therapeutic and diagnostic approaches. Overall, escaping these pathogens is a continuous journey, not the destination.

## Author contributions

Lingbing Zeng: Writing – Original Draft. Youjun Feng and Minggui Wang: Conceptualization, Supervision, Writing – Review and Editing.

## Consent for publication

All participants have provided informed consent for publication, and the authors have agreed to publish the manuscript in its current form.
